# Modeling Brittle Fractures in Epoxy Nanocomposites Using Extended Finite Element and Cohesive Zone Surface Methods

**DOI:** 10.3390/polym13193387

**Published:** 2021-10-01

**Authors:** John J. S. Biswakarma, Dario A. Cruz, Erich D. Bain, Joseph M. Dennis, Jan W. Andzelm, Steven R. Lustig

**Affiliations:** 1 Department of Chemical Engineering, Northeastern University, 360 Huntington Avenue, Boston, MA 02115, USA; biswakarma.j@northeastern.edu (J.J.S.B.); cruz.da@northeastern.edu (D.A.C.); jan.w.andzelm.civ@mail.mil (J.W.A.); 2 Engineering Systems, Inc., 10338 Miller Rd., Dallas, TX 75238, USA; erich.d.bain.civ@mail.mil; 3 Materials and Manufacturing Science Division, CCDC, U.S. Army Research Laboratory, 4600 Deer Creek Loop, Aberdeen Proving Ground, MD 21005, USA; joseph.m.dennis26.civ@mail.mil

**Keywords:** extended finite element method, cohesive zone surface, epoxy adhesives, three-point bending test

## Abstract

Linear elastic fracture modeling coupled with empirical material tensile data result in good quantitative agreement with the experimental determination of mode I fracture for both brittle and toughened epoxy nanocomposites. The nanocomposites are comprised of diglycidyl ether of bisphenol A cured with Jeffamine D-230 and some were filled with core-shell rubber nanoparticles of varying concentrations. The quasi-static single-edge notched bending (SENB) test is modeled using both the surface-based cohesive zone (CZS) and extended finite element methods (XFEM) implemented in the Abaqus software. For each material considered, the critical load predicted by the simulated SENB test is used to calculate the mode I fracture toughness. Damage initiates in these models when nodes at the simulated crack tip attain the experimentally measured yield stress. Prediction of fracture processes using a generalized truncated linear traction–separation law (TSL) was significantly improved by considering the case of a linear softening function. There are no adjustable parameters in the XFEM model. The CZS model requires only optimization of the element displacement at the fracture parameter. Thus, these continuum methods describe these materials in mode I fracture with a minimum number of independent parameters.

## 1. Introduction

Material ensembles such as adhered layers, fiber-reinforced polymer composites (FRPCs), and multi-layer coatings serve a wide range of industries including: construction, military, transportation, and aerospace [1,2]. In such industries, epoxy resins demonstrate remarkable utility due to their mechanical properties, chemical resistance, and sustainability. Epoxy resins are environmentally benign, a property that is attributed to curing processes that render a thermo-mechanically stable material. However, these curing processes yield potentially brittle materials, which is exemplified by the low strain-to-failure capacity of FRPCs [3,4]. There is hence an immediate need to develop efficient damage prediction tools for epoxy resins to guide the formulations of tough composites.

The field of theoretical continuum mechanics provides reliable techniques for simulating fracture processes. We assume that a linear elastic fracture mechanics (LEFM)-based approach is applicable for epoxy resins because the calculated size of their crack tip plastic zones was found to be small compared to common specimen dimensions and it is confined to a region close to the crack tip [2]. This study reports the use of two methods to model fracture processes available in the commercial software Abaqus (3DS—SIMULIA) [5]. The extended finite element method (XFEM) circumvents the need for mesh refinement in fracture process zones and conveniently inherits model definitions from conventional finite element methods [6]. XFEM provides an efficient framework for failure analysis when either an accurate description of in situ failure is available or a user-defined criterion is implemented to tame the over-prediction of failure conditions [7]. Cohesive zone models are also commonly used to model fractures in either an element-based or surface-based (CZS) implementation. Compared to XFEM, the cohesive zone methods are disadvantaged by having more stringent mesh dependencies for attaining convergence and by requiring predefined crack paths, which are difficult to ascertain in many realistic systems. In XFEM and CZS simulations, the cohesive traction–separation law (TSL) functions as the constitutive relationship that governs the crack initiation and propagation processes. Conveniently, TSLs can be customized to model a wide spectrum of constitutive equations that describe different classes of materials, such as glassy poly(methyl-methacrylate), concrete, and steel [8]. Recent studies using XFEM to model the failure of composite structures demonstrate the importance of selecting damage initiation and damage evolution criterion that are appropriate to the materials and systems studied, such as transverse plies of cross-ply carbon fiber/epoxy composites [9], glass fiber/polyvinylesters [7], and heavily crosslinked epoxy [4]. To the best of our knowledge, these methods have not been applied to the materials considered in this study.

We developed the methodologies herein with the intention of modelling the fracture processes exhibited by epoxy resin nanocomposites studied by Bain et al. [2]. We use the XFEM and CZS techniques to model the mode I fracture exhibited by samples of epoxy resins during the quasi-static single-edge notched bending (SENB) test. The resins are composed of diglycidyl ether of bisphenol A (Hexion Inc.) that has been cured with Jeffamine D-230 (Huntsman Chemical), a diamine propylene glycol oligomer with a molecular weight of 230 Da, and filled with varying concentrations of core-shell rubber nanoparticles MX-125 or MX-257 (Kaneka USA). The materials studied include two unfilled D230 epoxies, namely D230-1 and D230-2, and filled nanocomposites D230 + 1 wt% MX-125, D230 + 5 wt% MX-125, D230 + 1 wt% MX-257, and D230 + 5 wt% MX-257. The chemical structures of the two unfilled D230 materials are identical, except that the D230-1 was processed at a higher curing temperature and therefore has a higher glass transition temperature (*T_g_* = 99 °C) compared to the D230-2 (*T_g_* = 96 °C).

The predicted load-displacement behavior and mode I fracture toughness show satisfactory agreement with the experimental results for all simulations presented. The proposed methodologies are advantaged by circumventing user-defined subroutines and excessive non-physical hyperparameters. The fracture processes analyzed are induced by tensile stresses at the crack tip and hence uniaxial tensile data are sufficient for parameterizing the fracture model. Our models require three physical parameters, while the CZS model uses an additional fitting parameter, to accurately predict trends in load-displacement relationships and in the fracture toughness among the considered epoxy resins. All parameters are discussed in detail in Section 2. These simple LEFM-based methodologies can be generalized to predict the same trends among other classes of materials while relying on limited experimental data.

## 2. Materials and Methods

### 2.1. Fracture Model and System Layout

To model fracture in epoxy nanocomposites, we used constitutive models that include parameters formulated from the results of experimental SENB and uniaxial tensile tests. We conducted these tests according to the procedures adopted by Bain et al. [2]. Nominal stress-strain curves were determined for the epoxy resins from tensile tests that were conducted and post-processed according to ASTM standard D638-14 (see Figure S1, Supplementary Materials) [10]. However, we assumed that all materials behave as linear elastic solids and account for both plasticity and ductility by using TSLs to predict material damage and the separation between finite elements.

Fracture toughness quantifies the resistance of a sample to fracture. Determining the fracture toughness of a sample subjected to the SENB test requires measuring the load applied to the moving center pin of the three-point bending test apparatus at the critical point. The critical point of a SENB test is indicated at the maximum of the load-displacement curve, at which unstable fracture initiates upon further displacement of the center pin. The critical stress intensity factor K_IC_, also known as the fracture toughness, is calculated as [11]

(1) $$K_{\mathrm{IC}}=\frac{P\;}{{\mathrm{BW}}^{0.5}}f\left(\frac{a}{W}\right)$$

where P is the load applied at the critical point, B is the material depth or thickness, W is the material width, and a is the crack length. For materials obeying LEFM, the crack length is assumed to remain constant until the point of unstable fracture. The function $f\left(\frac{a}{W}\right)$ is defined according to ASTM standard D5045-14 [11].

**Figure 1:**
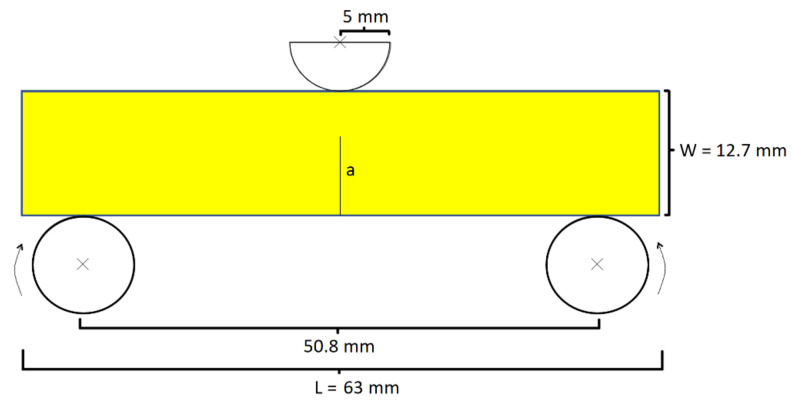
Configuration of the simulated SENB test apparatus within which the stationary bottom pins are rotatable.

One goal of this study is to validate using LEFM assumptions in modelling the quasi-static SENB test. Figure 1 presents the configuration of a SENB test simulated using the Abaqus software. The dimensions of all modeled samples comply with the guidelines for the SENB test for plastic materials established by ASTM standard D5045-14 [11]. We performed simulations for six epoxy resin samples possessing different initial crack length a. Note that the initial crack of a sample is modeled as a seam line at which the adjacent material surfaces are unbound to each other. The dimensions of all samples modeled in this study are presented in Table 1, except for the width W, which was assumed to be 12.7 mm for each sample. This is a reasonable assumption because the experimental measurements differ from this dimension by 1% at most. Note that Figure 1 does not show the depth of the sample because all samples were modeled with an implicitly in-plane depth B. Furthermore, these samples’ thicknesses are at least 50x the theoretical plastic zone radius size and thus the crack tip region is in a state of plane strain deformation [2,11]. Therefore, our assumption that the samples are in plane strain deformation during the SENB test is justified.

In this study, the tensile yield stress is used to inform the criterion for initiating fracture between finite elements. This is justified by considering that the yield stress of a material represents the stress at which significant plastic deformation occurs and that concentrated regions of both stress and plastic strain form ahead of a crack tip when a load is applied in the SENB test. The yield stress of each material is assumed to be the local maximum stress from the experimental stress-strain curves. The resulting yield stresses are listed in Table 2. In the case of unfilled D230, the reported yield stress is the average of the measured yield stresses of the D230-1 and D230-2 materials. For each material the yield stress reported in Table 2 is included as a parameter in the TSLs.

Fracture data for epoxy resins obtained from experimental SENB tests were used to formulate criteria for predicting fracture initiation and crack propagation. Poisson’s ratio and an effective elastic modulus were the physical parameters used to define the materials studied. A Poisson’s ratio of 0.35 was assumed for every material to be consistent with experiments conducted by Bain et al. [2]. For each epoxy system, the experimentally determined mode I fracture toughness K_IC_ and critical strain energy release rate G_IC_ (Table 3) were used to determine [11] the effective elastic modulus E_eff_.

(2) $$E_{\mathrm{eff}}=\frac{\left(1-v^{2}\right)K_{\mathrm{IC}}^{2}}{G_{\mathrm{IC}}}\;$$

This definition of E_eff_ sufficiently describes brittle isotropic solids under plane strain deformation [12]. G_IC_ informs the crack propagation (damage evolution) because it is the rate of energy change corresponding with the increase of surface area that occurs during mode I crack growth. Note that Equation (2) provides a lower bound for E_eff_ because K_IC_ represents a lower limiting value of the mode I fracture toughness [11]. The experimental fracture test results and calculated E_eff_ are provided for each material in Table 3. These physical parameters were used to model the SENB test for each material considered in this study.

### 2.2. Surface-Based Cohesive Zone Model

The cohesive zone model incorporates discontinuities in materials by forming a cohesive damage zone that allows for the modelling and analysis of interfacial fracture [13]. The CZS method is often used to study delamination of surfaces [14], unlike the element-based cohesive zone model approach frequently used to study adhesive layers [15]. In our CZS simulations, we define contact pairs between material interfaces to represent the cohesive zone, as performed by Li et al. [16]. Damage evolution (crack propagation) is governed by a TSL that represents the functional relationship between the traction *T_r_* and displacement between nodes at the crack tip *Δ*. Fracture initiates after stresses at elements satisfy a pre-defined criterion and the progress in separation between elements is governed by a softening function. The quadratic nominal stress (QUADS) and maximum principal stress (MAXPS) fracture initiation criterion were used for CZS and XFEM simulations, respectively. A softening function can be selected to better simulate the expected brittle or ductile type of fracture process [17]. Furthermore, the fracture processes exhibited by similar materials tend to be well-described by TSLs using the same class of softening functions. Our work adapts a generalization of trapezoidal softening functions, which are often used to model ductile materials [18]. Commonly used softening functions are defined in terms of hyperparameters that introduce additional fitting capabilities.

**Figure 2:**
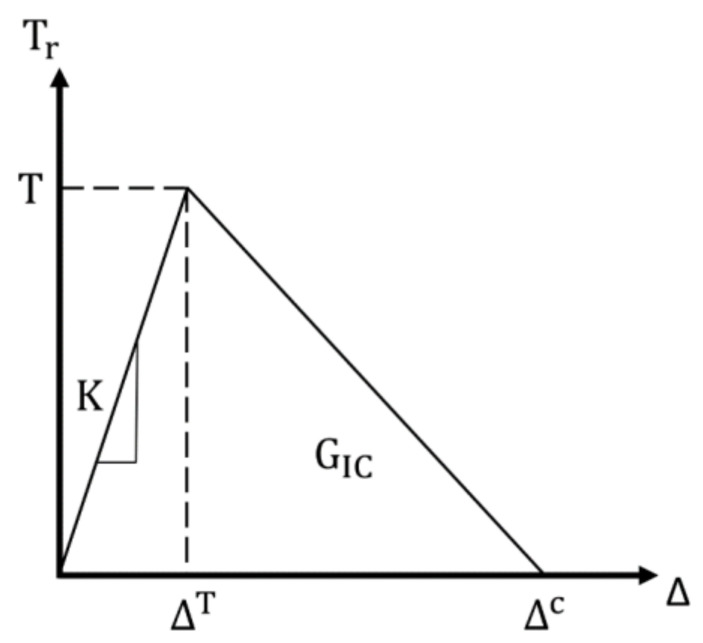
The linear traction–separation law (TSL) used to model damage initiation and evolution of the mode I fracture. This plot represents the c = 1 case of the truncated linear TSL.

This study assesses the applicability of a truncated linear softening function in modeling the brittle fracture of epoxy nanocomposites. This approach has been reviewed previously for other materials [8]. Considering mode I fracture was being modelled, the area enclosed by the softening function and loading portion of the TSL (Figure 2) was equivalent to the critical strain energy release rate G_IC_ [11]. Furthermore, the interface stiffness *K* determines the initial slope of the TSL. We explored the proper selection of values for the stiffness parameter *K* as well as its connection to the TSL [19]. Additionally, a softening function can be adjusted to model the type of material in question more accurately [8,19]. The general form of the truncated linear TSL is defined as,

(3) $$T_{r}=\left\{\begin{array}{c} K\Delta ,\;\;\;\;\;\;\;\;\;\;\;\;\;\;\;\;\;\;\;\;\;\;\Delta \leq \Delta^{T}\;\\ -\frac{cT\left(\Delta -\Delta^{T}\right)}{\Delta^{c}-\Delta^{T}}+T,\;\;\;\Delta^{T}<\Delta \leq \Delta^{c}\\ 0,\;\;\;\;\;\;\;\;\;\;\;\;\;\;\;\;\;\;\;\;\;\;\;\;\Delta >\Delta^{c} \end{array}\right.$$

where the hyperparameter *c* is a geometrical descriptor that determines the point at which the softening function is truncated, Δ*^T^* is the displacement at fracture initiation $\left(\frac{T}{K}\right)$, and Δ*^c^* is the displacement at complete fracture. At the region of TSL in which no damage is done to the material (0 < Δ *<* Δ*^T^*), the displacement between nodes is miniscule and entirely reversible. The peak traction *T* is used to define the QUADS parameter in Abaqus simulations and thus it informed the damage initiation criterion of our CZS model. The QUADS parameter is calculated as the sum of quadratic ratios between the measured stresses and peak nominal stresses in three dimensions, which includes tractions normal to the crack interface and in two shear directions. We assigned the tensile yield stress as the peak nominal stress of each dimension.

The area enclosed by the TSL is constrained to be the value G_C_ [8] and thus for any valid value of *c* (0 *< c* ≤ 1), the TSL needs to be adjusted to uphold this constraint. Furthermore, the interfacial strength *T* (i.e., the maximum traction value of the TSL) is assumed to be equivalent to the tensile yield stress of a material. Thus, the displacement Δ*^c^* can be calculated using *c*, stiffness *K*, and traction *T* as

(4) $$\Delta^{c}=\left(\frac{2G_{C}}{T}-\frac{T}{K}\right)\left(\frac{1}{2-c}\right)+\frac{T}{K}$$

For the truncated linear softening function, the damage variable *D* is calculated as:

(5) $$D\left(\Delta \right)=\left\{\begin{array}{c} c\frac{\Delta -\Delta^{T}}{\Delta^{c}-\Delta^{T}},\;\;\;\;\;\Delta^{T}<\Delta <\Delta^{c}\\ 1,\;\;\;\;\;\;\;\;\;\Delta \geq \Delta^{c} \end{array}\right.$$

where the input data are included in Abaqus simulations as tabular values of *D* as a function of Δ *−* Δ*^T^*. Multiple displacement values between Δ*^T^* and Δ*^c^* with corresponding *D* values are necessary to include in the definition of the TSL to obtain well-converged results. Elements along the crack that are separated such that Δ*^T^* < Δ < Δ*^c^* are partially damaged (0 < *D* < 1). Displacements less than Δ*^T^* indicate that the relevant elements are undamaged (*D* = 0), whereas displacements greater than or equal to Δ*^c^* indicate that the relevant elements are separated and completely damaged (*D* = 1). Elements along the initial crack do not experience cohesive interactions and are considered completely damaged.

In implementing the truncated linear TSL, we observed that the hyperparameter of *c* = 1 led to the most accurate prediction of fracture data for all the materials considered. We have decided to report only the results obtained from this case because we are primarily interested in the ability of these methods to accurately model the fracture processes of epoxy resins. Thus, we must emphasize that a linear TSL was used in all simulations for the data reported in this study.

Table 4 provides all parameters that define the damage initiation and evolution criteria for the CZS simulations conducted. Note that in the case of CZS simulations, the high stress concentrations surrounding the crack tip (the fracture process zone) necessitate fine mesh resolutions to accurately model fracture processes. It is observed that the quality of such simulations depends on the number of mesh elements within the fracture process zone [19]. The stiffnesses *K* were selected to approximately fit the experimental fracture data for each material.

### 2.3. Extended Finite Element Method

The XFEM method adds enrichment functions to the conventional finite element mesh to allow discontinuities in the displacement field ***u***(the crack domain *Γ_c_*) and singularities that occur at crack tips [4,5]. The discretized displacement field ***u****^h^* in XFEM is thus written as

(6) $$u^{h}\left(x\right)=\sum_{i\epsilon I}u_{i}N_{i}\left(x\right)+\sum_{j\epsilon J}N_{j}\left(x\right)H\left(x\right)b_{j}+\sum_{k\epsilon K}N_{k}\left(x\right)\left(\sum_{l=1}c_{k}^{l}F_{l}\left(x\right)\right)$$

where x denotes material coordinates, *I* is the set of all nodes in the mesh, *J* is the set of crack tip nodes, *K* is the set containing related enrichment nodes, $u_{i}$ is the displacement field degrees of freedom at the node *i*, $N_{n}\left(x\right)$ is the shape function associated with the node *n,* $H\left(x\right)$ is the step function, and $b_{j}$ is the nodal enrichment degrees of freedom which represent the amount of displacement in the crack line. The last expression in Equation (6) is the crack enrichment term, which contains the crack tip asymptotic functions $F_{l}\left(x\right)$, and the crack tip-enriched nodal degrees of freedom $c_{k}^{l}$, which represent the displacement in the crack tip [20].

In our XFEM simulations, the fracture process was governed by a linear TSL. For each material, the stiffness *K* was equivalent to the effective elastic modulus (Table 3) divided by 1 mm, which is a default setting available in the Abaqus software for XFEM simulations. The critical strain energy release rate was calculated using the Benzeggagh and Kenane approach [21], while the energy released per unit area during pure mode I fracture was equivalent to G_IC_ (Table 3). The tensile yield stress, converted to true stress, was assigned as the MAXPS fracture initiation criterion parameter for all XFEM simulations. The progress of a fracture was monitored using the damage variable STATUSXFEM in Abaqus simulations, which changes from 0 to 1. A STATUSXFEM value of 1 indicates a complete separation of crack face elements.

### 2.4. Simulation Parameters

Significant computational model parameters including details on meshing conditions are listed in Table 5. In both the XFEM and CZS models, we assigned a uniform mesh to the bulk material, and in the CZS model, we included a fine mesh along the predefined crack path. In all simulations, we defined the crack as a line segment with an infinitesimally small width and the initial length was observed in the experimental SENB test. Note that we simulated a version of the SENB test that is designed such that the bottom pins are rotatable with as minimal friction as possible [11]. We performed simulations with and without rotatable pins, and did not observe significant differences in results. We have presented data obtained from modelling the two adjacent pins as explicitly rotatable because this feature introduced additional control over the emulation of experimental conditions. Furthermore, the sliding contact between all pins and material surfaces had a friction coefficient of 0.1 and the punch (top pin) was modeled as a rigid analytical surface. We also observed that the prediction of the critical point was insensitive to the material density. This implies that the mode I fracture toughness predicted for each material is independent of material density and thus the assumption that every material possessed the same density is acceptable. Hence, we assumed that the density of each material was equivalent to the experimentally measured density of unfilled D230 resin measured as 1.156 × 10^−9^ tonne/mm^3^ (1.156 g/cm^3^).

**Figure 3:**
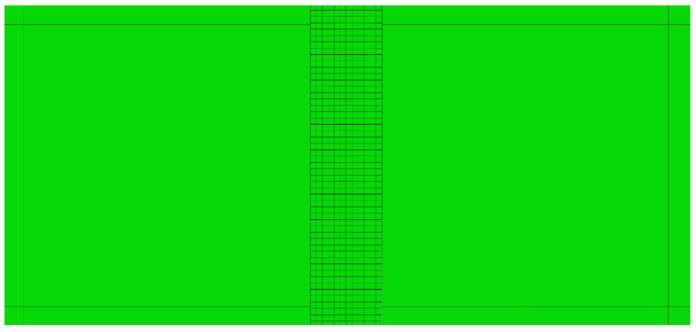
Close-up image of the mesh of the sub-laminate layer that was used in the CZS simulations.

Square mesh elements were used in each simulation. Mesh elements in the bulk material had edge lengths of 0.05 mm in the XFEM simulations and 0.2 mm in the CZS simulations. Extensive validation of the mesh design was performed for the XFEM model, documenting that the selected mesh yields converged results (see Figures S2 and S3). The CZS simulations included a sub-laminate layer containing smaller mesh elements, as depicted in Figure 3. This adjustment was required to obtain convergent CZS simulations and better resolution for monitoring stress distributions near the crack tip during the fracture process. We found that it was necessary for the sub-laminate layers to be wider than the length of the plastic zone ahead of the crack tip predicted under plain strain deformation in the Tresca yielding criteria [22] for unfilled D230:

(7) $$r_{p}=\frac{1}{2\pi }{\left(\frac{K_{IC}}{\sigma_{ys}\;}\right)}^{2}$$

where $\sigma_{ys}$ is the tensile yield stress (Table 2) and K_IC_ is the mode I fracture toughness (Table 3) of unfilled D230. The predicted plastic zone length for unfilled D230 was 47 μm and hence our CZS simulations contained 50 μm sub-laminate layers along each side of the crack. Using the predicted plastic zone of unfilled D230 as a constraint for the sub-laminate layer width is justified because our methodologies assume that all materials are brittle linear elastic materials. The square mesh elements within the sub-laminate layers were assigned edge lengths of 0.005 mm, which was found to be sufficiently small for convergent simulations. Furthermore, we found that smaller sub-laminate mesh elements led to a numerical stability that was exhibited as hour-glassing.

## 3. Results and Discussion

**Figure 4:**
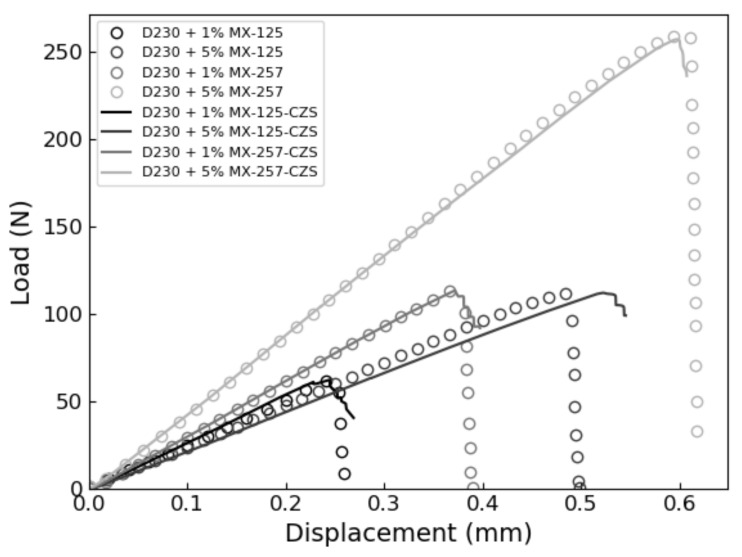
CZS-predicted SENB test load-displacement curves (solid lines) benchmarked against experimental data for each nanocomposite resin.

**Figure 5:**
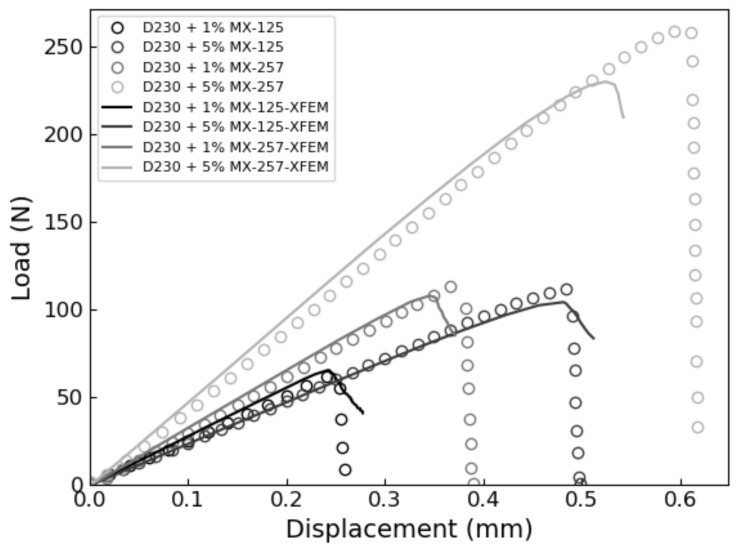
XFEM-predicted SENB test load-displacement curves (solid lines) benchmarked against experimental data for each nanocomposite resin.

**Figure 6:**
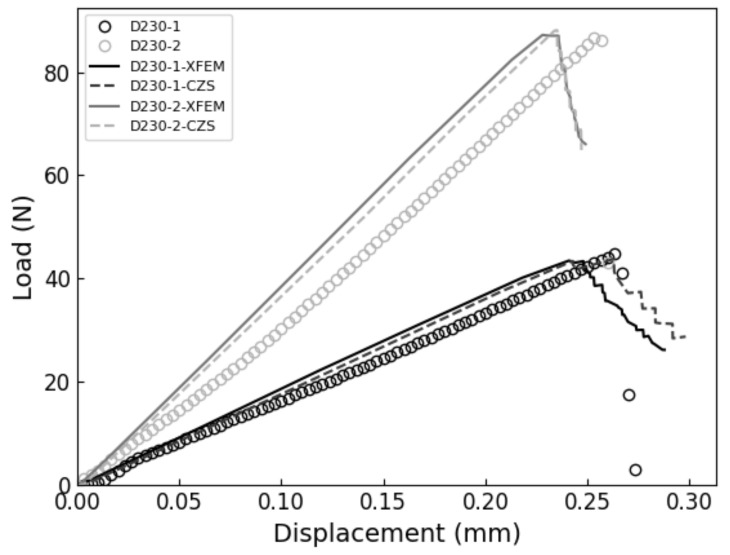
Experimental vs. XFEM and CZS-predicted SENB test load-displacement curves for unfilled D230 resins.

**Figure 7:**
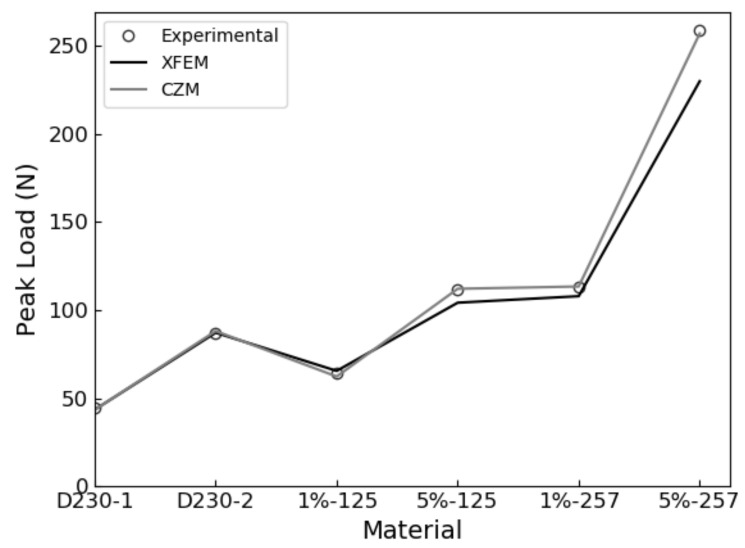
Experimental vs. predicted maximum loads P_max_ for each material.

**Figure 8:**
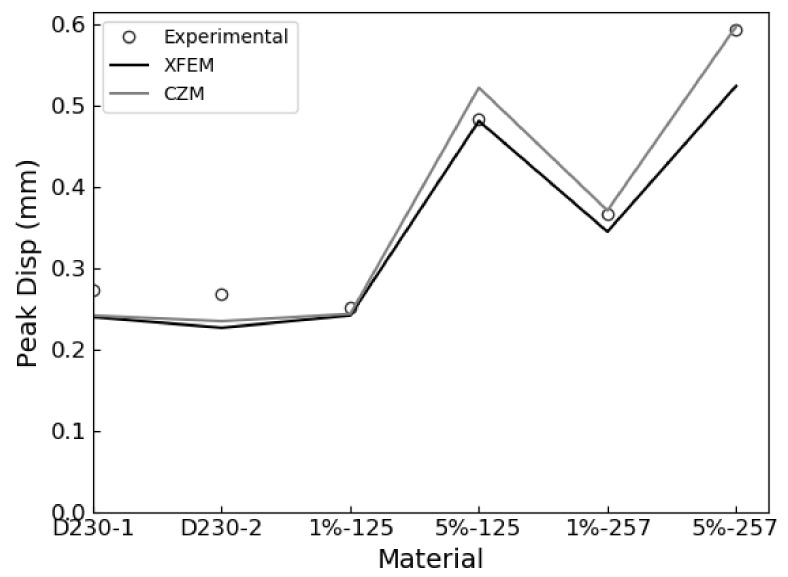
Experimental vs. predicted load-line displacements (LLDs) d_c_ for each material.

**Figure 9:**
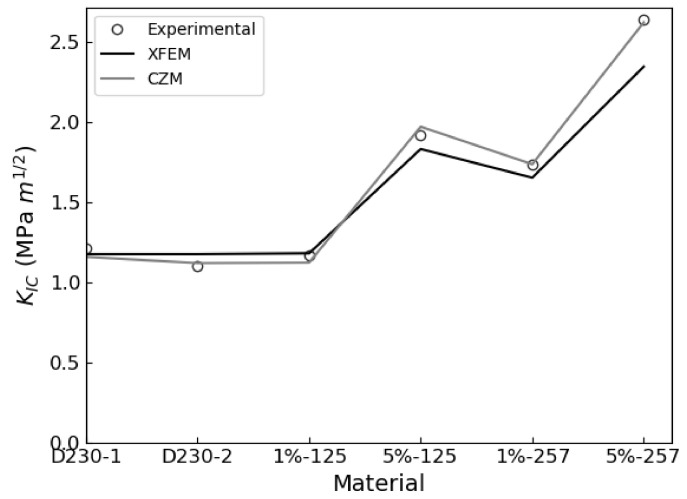
Experimental vs. predicted mode I fracture toughness K_IC_ for each material.

The XFEM and CZS methods were used to simulate the brittle mode I fracture of six epoxy nanocomposites that occurs during the quasi-static SENB test. From each simulation, a load-displacement plot was generated and compared with experimental results. We identified the critical load that occur during the SENB test when there is complete separation between elements at the crack tip and where unstable fracture initiates. The critical point is identified when the parameter STATUSXFEM attains a value of 1 during XFEM simulations or, in the case of the CZS simulations, when the separation between elements at the crack tip become larger than the Δ*^c^* value listed in Table 4. The load applied to the center pin at the critical point is used to calculate the mode I fracture toughness K_IC_ according to Equation (1). The maximum load and corresponding load-line displacement (LLD) are identified and labeled as P_max_ and d_c_, respectively, to compare with experimental results. The results are summarized in Table 6 and load-displacement curves predicted using XFEM and CZS are shown in Figure 4, Figure 5 and Figure 6 with the experimental data. For each material, these simulations approximately predict the critical point of the SENB test and the mode I fracture toughness K_IC_. One can see that the simulations follow the trends observed in the experiments, as shown in Figure 7, Figure 8 and Figure 9.

These simulations successfully predicted that brittle fracture processes occur in each material studied when subjected to the SENB test. The proposed methodologies quantitatively predict the critical point exhibited in the SENB test. Therefore, a linear elastic constitutive model that accounts for fracture using a linear TSL for modelling fracture is sufficient for simulating brittle fracture processes exhibited by epoxy resins. Furthermore, the yield stress determined from the experimental uniaxial tensile tests suffices for defining the criterion for the initiation of brittle fracture. Overall, there is good quantitative correlation between the experimental results and both XFEM and CZS predictions for both the unfilled and rubber-filled epoxy materials. This approach does not describe complex micromechanical toughening mechanisms and plastic deformations at the crack tip, but rather offers a sufficient approximation at a continuum level to predict fracture for the class of epoxies studied.

Error propagation analysis indicates that the experimental data were adequately reproduced by the XFEM and CZS methods. Percent errors between the simulated and experimental results presented in Table 6 were less than 10% in all cases other than the XFEM simulation for D230 + 5 wt% MX-257, which attained values up to 11.6%. In this case, the expected uncertainty for predictions was approximately 12%, which is the quadrature sum of the relative standard deviations of the experimentally determined tensile yield stress and elastic modulus. Our model inherits this uncertainty due to incorporating the tensile yield stress in the fracture initiation criteria and due to using both the experimentally determined mode I fracture toughness K_IC_ and critical strain energy release rate G_IC_ in Equation (1) to calculate the effective elastic modulus E_eff_ [11]. Note that this argument assumes that the predicted peak load, critical displacement, and mode I fracture toughness change linearly with respect to the tensile yield stress and elastic modulus, which is reasonable for small deviations. In general, our predictions are within 2.4% of the uncertainties derived from the standard deviations of the experimentally determined yield stresses and elastic moduli. Note that discrepancies between our CZS and XFEM-predicted results are not attributed to the different degrees of convergence because our simulations are fully converged with respect to the meshes used. The XFEM and CZS models also have different degrees of freedom to fit experimental data. In the CZS simulations, the stiffness parameter *K* was adjusted to enable closer agreement with experimental results. In the XFEM model, the stiffness parameter was equal to the effective elastic modulus and was not adjusted to fit the experimental data. This difference between these models contributes to the discrepancies of their predictions. Overall, our models sufficiently model the SENB test and predict the corresponding fracture properties for each material considered.

Our models tended to predict the critical loads more accurately than the critical LLDs. On average, the error of the predicted critical loads was approximately 4.5 times smaller than the error of the predicted critical LLDs. In our simulations the pins are treated as rigid, non-deformable objects. Therefore, the system compliance observed in experimental SENB tests, which additively contributes to the measured LLDs, did not affect our simulations [11]. Our models also did not account for the formation of biaxial stress states and material deformations that formed in the crack plane, i.e. the face formed by the crack length and width [23]. This resulted from our assumption that each sample exhibits a 2D homogeneous plane strain condition. SENB tests modelled under this assumption tend to overestimate the material stiffness [24]. Consequently, our underestimation of critical LLDs is attributed to the assumption that the samples exhibit the 2D homogeneous plane strain condition.

**Figure 10:**
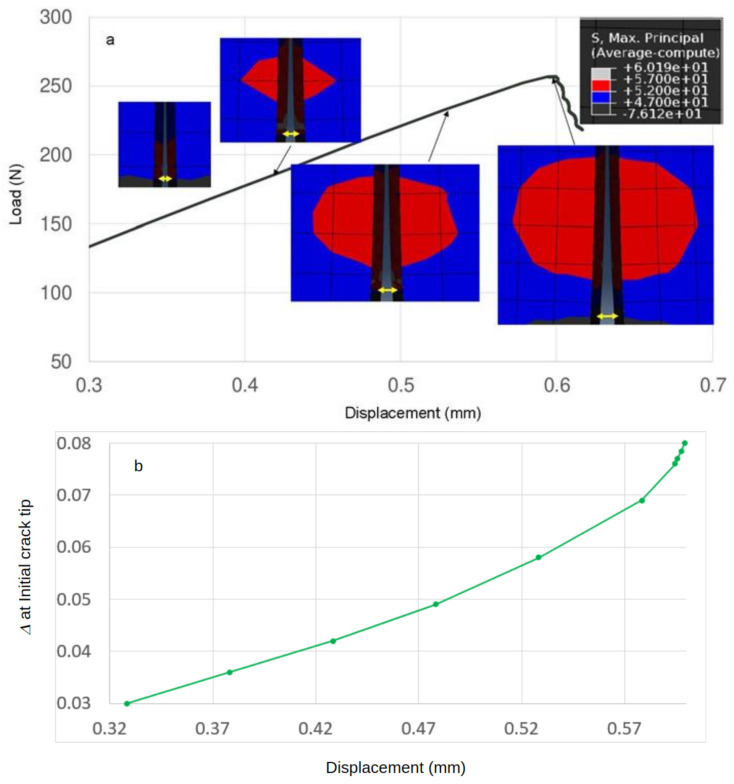
CZS prediction of the fracture process for D230 + 5 wt% MX-257. (**a**) Load vs. LLD, with the corresponding maximum principal stress (MAXPS) contours shown at various points. Yellow arrows indicate the degree of separation between the nodes at the initial crack tip. (**b**) Separation between crack faces at the crack tip vs. LLD.

We calculated principal stress contours and monitored the separation between the elements at the initial crack tip. Figure 10 includes results obtained from analysis of the CZS simulation for D230 + 5 wt% MX-257. Figure 10a shows a load-displacement curve with superimposed contours of principal stress that enclose the fracture process zone. Prior to the initiation of the unstable fracture, the fracture process zone increased in size with the applied load, reaching a maximum value of 257.0 N (Table 6). The size of the fracture process zone remained approximately constant as the crack continued to progress. Although we did not explicitly employ plasticity models in the simulations, the formation of the fracture process zone and plastic zone that formed around the crack tip correspond to the increased toughness of the nanocomposites. We also found that the maximum value of the principal stress was attained close to the critical point of the load-displacement curve. The separation between the crack faces at the initial crack tip increased with the applied load, but the crack did not propagate until the critical point was reached. The critical point is reached when the distance between elements at the initial crack becomes larger than the Δ*^c^* value of 0.0791 mm (Figure 10b). The corresponding load of 256.9 N was very close to the observed maximum load of 257.0 N. Using the value of 256.9 N to calculate the K_IC_ value according to Equation (1) accurately predicts the experimental result, as shown in Table 6. The separation between elements at the crack tip increased non-linearly (Figure 10b) and fracture occurred at d_c_ = 0.598 mm (Table 6). We also calculated the size of the fracture process zone at the critical point for each polymer studied. We observed that the size of the fracture process zone increased with the toughness of the polymer nanocomposite system. Detailed analysis and quantitative assessment of the fracture process zone and plastic zone would require a much denser mesh than employed in this study.

While the current approach effectively replicates the increased toughness of nanocomposites, which is well known to occur due to complex micromechanical plastic deformation processes near the crack tip [25], these models do not address this phenomenon directly. A more detailed approach could entail using a hyper-elastic-based constitutive model that predicts plastic deformations at the crack tip, particularly for rubber-filled materials [26]. One could also directly account for the plasticity in the constitutive models used for these materials. Material plasticity could be indirectly treated by accounting for plastic strains that occur during the uniaxial tensile test [27]. The deformed cross-sectional areas of tensile test samples, which are required to exactly calculate the true stress, are unavailable and thus stress values beyond the ultimate yield stress could be extrapolated [28]. Measurements of the transverse strain in one or two dimensions using high resolution digital image correlation of uniaxial tensile tests would allow for the more direct measurement of Poisson’s ratio and would enable the use of complex empirical plasticity models [29]. The accuracy of our predictions could also be improved by optimizing the yield stress, which is used to define the fracture initiation criterion. Optimizing the offset that is used to determine the yield stress of each sample from the experimental tensile data considered would account for the varying degrees of plasticity among the materials considered [10,30]. Note that the purpose of this study is to validate continuum methods using a minimal number of fitted parameters and hence we did not pursue this additional optimization.

## 4. Conclusions

This study demonstrates two LEFM-based methodologies in modelling the brittle mode I fracture of epoxy resin nanocomposites observed during the quasi-static SENB test. The XFEM and CZS methods are comparably robust and successful in modelling the fracture processes exhibited by the epoxy resins considered. These methods accurately predict the critical point of the SENB test, which enables the accurate determination of mode I fracture toughnesses K_IC_. This overall agreement between the predicted and experimental values justifies applying the present methods in fracture modeling of both brittle and toughened epoxy resins.

We have proposed methodologies that are well advantaged by their simple parameterization and reliance on minimal independent empirical data. Damage initiates upon stresses at the crack tip, attaining the yield stress, which is determined from experimental uniaxial tensile tests. This approximation is sufficient for all epoxy systems considered in this study. A general form of truncated linear TSL was considered and the best results were obtained using an entirely linear formulation of the damage model. There are no adjustable parameters of the XFEM model and the CZS model requires optimization of only the stiffness parameter K, which defines the displacement between elements at complete fracture Δ*^c^*. The demonstrated predictive capability of the CZS model asserts that this parameterization is consistent. The displacement Δ*^c^* was determined through optimization of the TSL and was used to predict the onset of complete fracture between nodes at the simulated crack tip. The corresponding critical load was used in the calculations of the fracture toughness K_IC_. Calculating stress contours at the crack tip enables monitoring the progression of the unstable brittle fracture process.

This research study is concerned with the validation of continuum methods for modelling fracture properties of epoxy resins that depend on minimal experimental parameters. Neither method used in this study required extensive customization or detailed plasticity modeling. However, accounting for toughening mechanisms at the microscale was not undertaken in this work [31,32] and is left for further developments. Future work may also involve refining the TSL by either further optimizing the softening function or by using an established inverse method to fit a TSL to experimental data [33]. Our simulations also do not account for complex phenomena such as the occurrence of modes of plastic deformation, crack tip-blunting, and explicit dependencies on both crack tip position and velocity.

## Figures and Tables

**Table 1:** Dimensions of the epoxy resin samples analyzed with the single-edge notched bending (SENB) test. All samples tested had a length of 63 mm.

Material	Width, W (mm)	Depth, B (mm)	Initial Crack Length, a (mm)
D230-1	12.74	6.00	8.26
D230-2	12.74	6.10	5.56
D230 + 1 wt% MX-125	12.57	6.10	6.85
D230 + 5 wt% MX-125	12.71	6.10	6.85
D230 + 1 wt% MX-257	12.73	6.21	6.40
D230 + 5 wt% MX-257	12.80	6.11	4.57

**Table 2:** Yield stresses of each epoxy resin obtained from experimental uniaxial tensile tests. These values are converted to true stresses, which are required for Abaqus input files.

Material:	D230	D230 + 1 wt% MX-125	D230 + 5 wt% MX-125	D230 + 1 wt% MX-257	D230 + 5 wt% MX-257
Yield stress (MPa):	70.7	66.0	59.0	62.0	57.0

**Table 3:** Fracture properties of epoxy resins determined from experimental SENB tests. The G_IC_ and K_IC_ values are used to calculate E_eff_ according to Equation (2).

Material	G_IC_ (kJ/m^2^)	K_IC_ (MPa∙m^1/2^)	E_eff_ (MPa)
D230-1	0.394	1.215	3281.1
D230-2	0.406	1.105	2640.1
D230 + 1 wt% MX-125	0.428	1.167	2796.1
D230 + 5 wt% MX-125	1.431	1.963	2361.6
D230 + 1 wt% MX-257	0.946	1.738	2800.1
D230 + 5 wt% MX-257	2.362	2.640	2588.9

**Table 4:** Parameters for surface-based cohesive zone (CZS) simulations using a linear TSL (c = 1).

Material	D230-1	D230-2	D230 + 1 wt% MX-125	D230 + 5 wt% MX-125	D230 + 1 wt% MX-257	D230 + 5 wt% MX-257
Δ*^c^* (mm)	0.0105	0.0108	0.0121	0.0462	0.0290	0.0791
*K* (MPa/mm)	110000	100000	80000	25000	40000	15000

**Table 5:** Parameters used in all CZS and extended finite element method (XFEM) simulations.

Simulation Parameter	Value
2D element type	CPE4R
Hourglass control type	Stiffness
Hourglass parameter	0.5
# nodes in bottom pins	100
Contact type	Hard

**Table 6:** Fracture properties for each epoxy resin. K^IC^ was calculated according to Equation (1). Percent error was calculated for the predicted model relative to the experimental results.

Case	P_max_ (N)	% Error	d_c_ (mm)	% Error	K_IC_ (MPa∙m^1/2^)	% Error
D230-1						
Experiment	44.8		0.263		1.215	
CZS	43.8	−2.23	0.263	0	1.160	−4.53
XFEM	43.5	−2.90	0.241	−8.37	1.179	−2.96
D230-2						
Experiment	86.7		0.253		1.106	
CZS	88.1	1.61	0.235	−7.11	1.122	1.45
XFEM	87.2	1.15	0.228	−9.88	1.113	0.633
D230 + 1 wt% MX-125						
Experiment	64.6		0.252		1.167	
CZS	62.4	−3.53	0.244	−3.17	1.105	−5.31
XFEM	65.5	1.39	0.243	−3.57	1.183	1.37
D230 + 5 wt% MX-125						
Experiment	111.5		0.483		1.923	
CZS	112.1	0.538	0.523	8.28	1.973	2.60
XFEM	104.2	−6.55	0.481	−0.414	1.834	−4.63
D230 + 1 wt% MX-257						
Experiment	113.3		0.367		1.737	
CZS	113.5	0.177	0.372	1.36	1.738	0.0576
XFEM	107.9	−4.77	0.345	−5.99	1.654	−4.78
D230 + 5 wt% MX-257						
Experiment	258.6		0.594		2.640	
CZS	257.0	−0.619	0.598	0.673	2.623	−0.644
XFEM	230.0	−11.1	0.525	−11.6	2.347	−11.1

## Data Availability

The data presented in this study are available upon request from the corresponding author.

## Supplementary Materials

The following are available online at https://www.mdpi.com/article/10.3390/polym13193387/s1, Figure S1: Engineering stress-strain curves determined from uniaxial tensile tests for each material, Figure S2: Convergence of critical load in XFEM simulations with respect to mesh size, Figure S3: Convergence of critical load-line displacement in XFEM simulations with respect to mesh size.

## Abbreviations

CZS	Surface-based cohesive zone method
FRPC	Fiber-reinforced polymer composite
LEFM	Linear elastic fracture mechanics
LLD	Load-line displacement
MAXPS	Maximum principal stress
QUADS	Quadratic nominal stress
SENB	Single-edge notched bending
TSL	Traction-separation law
XFEM	Extended finite element method
