# Supplementary material for: Modeling Brittle Fractures in Epoxy Nanocomposites Using Extended Finite Element and Cohesive Zone Surface Methods
Source: Polymers (Basel). 2021 Oct 1;13(19):3387. doi: 10.3390/polym13193387 (PMC8512390; doi:10.3390/polym13193387)
Supplement: Supplementary file 1 [file polymers-13-03387-s001.zip › polymers-1313104-supplementary.pdf]

polymers

Supplementary Material: Modelling Brittle Fracture of Epoxy Nanocomposites using Extended Finite Element and Cohesive Zone Surface Methods

John J. S. Biswakarma, Dario A. Cruz, Erich D. Bain, Joseph M. Dennis, Jan W. Andzelm and Steven R. Lustig

Supplementary Information 1: Uniaxial tensile test results

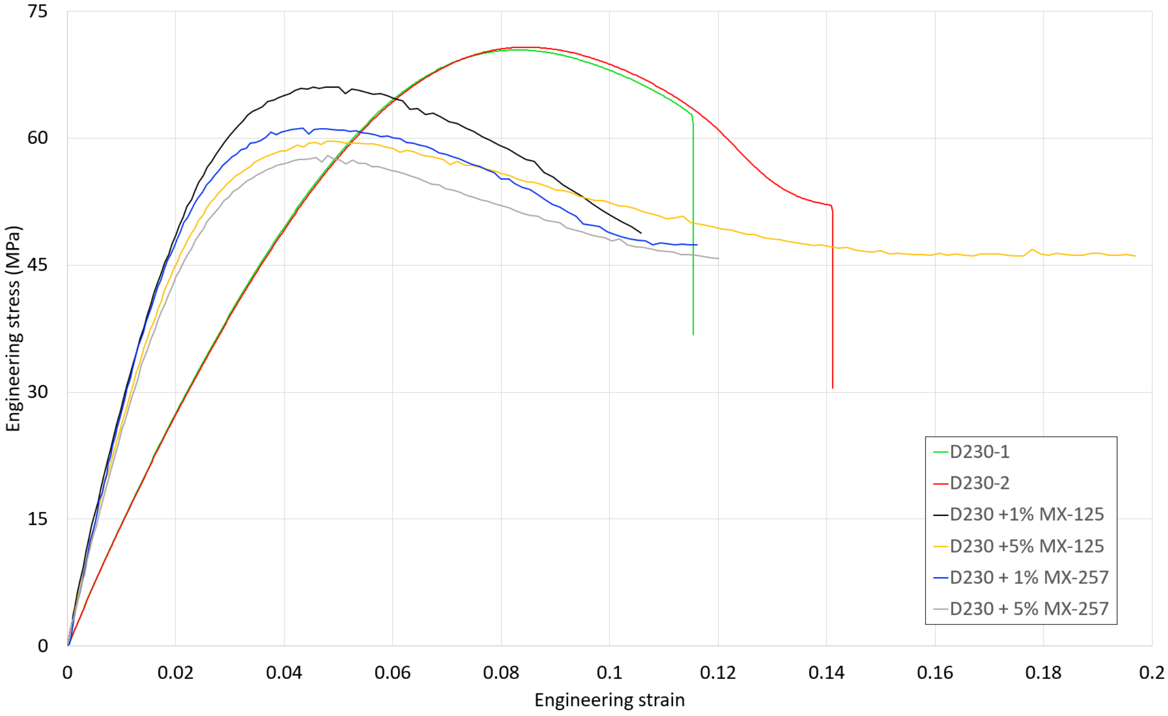

Figure S1. Engineering stress-strain curves determined from uniaxial tensile tests for each material.

Supplementary Information 2: Mesh sensitivity of XFEM simulations

XFEM predictions of P_{max} and d_c are well-converged with respect to the mesh density as shown in Figures S2 and S3. The "Unfilled" material is a D230 resin possessing material properties and exhibiting fracture and tensile behavior like that of the D230-1 and D230-2 resins.

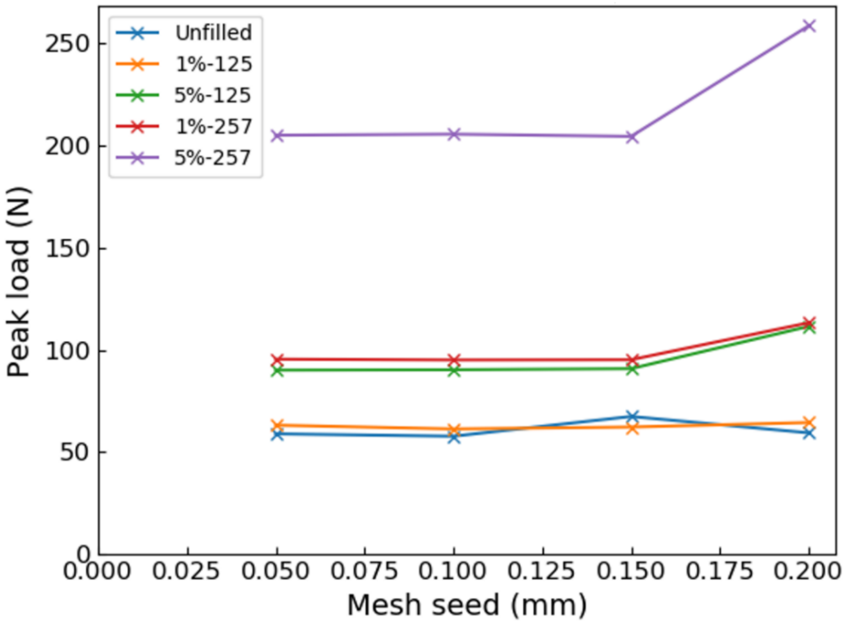

Figure S2. Convergence of critical load in XFEM simulations with respect to mesh size.

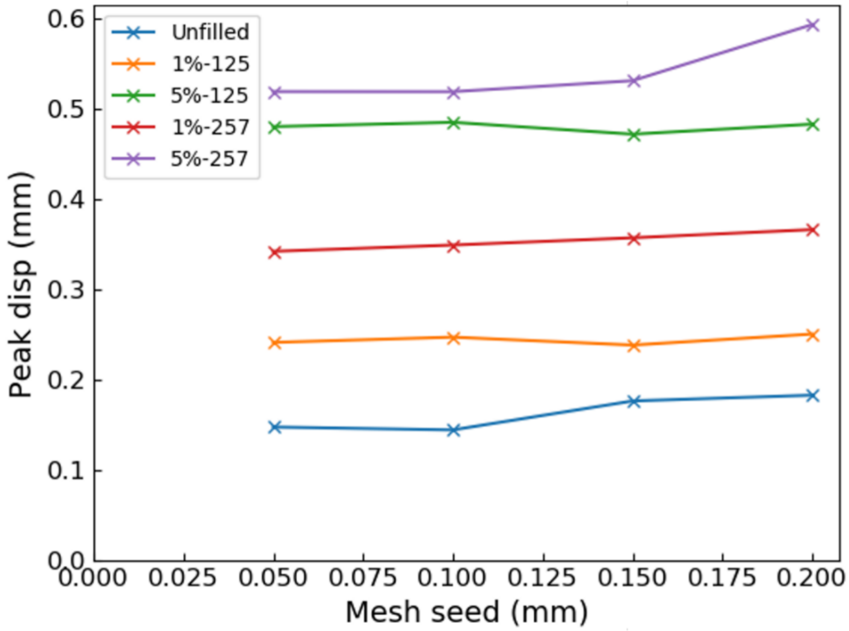

Figure S3. Convergence of critical load-line displacement in XFEM simulations with respect to mesh size.
